# Supplementary material for: TiO2 Nanoparticle/Polyimide Nanocomposite for Ultrahigh-Temperature Energy Storage
Source: Nanomaterials (Basel). 2022 Dec 15;12(24):4458. doi: 10.3390/nano12244458 (PMC9780931; doi:10.3390/nano12244458)
Supplement: Supplementary file 1 [file nanomaterials-12-04458-s001.zip › nanomaterials-1995853-supplementary.pdf]

Supporting Information

TiO_2 nanoparticles/polyimide nanocomposite for ultrahigh-temperature energy storage performance

Xinrui Chen[1, 2], Wenbo Zhu[1], Jianwen Chen[2, *], Qing Cao[1, 2], Yingxi Chen[1, 2], Dengyan Hu[1, 2]

[1]*School of Mechatronics Engineering and Automation College, Foshan University, Foshan, 528200, P. R. China*

[2]*School of Electronic and Information Engineering, Foshan University, Foshan, 528200, P. R. China*

*To whom correspondence should be addressed. E-mail: iamjwen@126.com

Figures

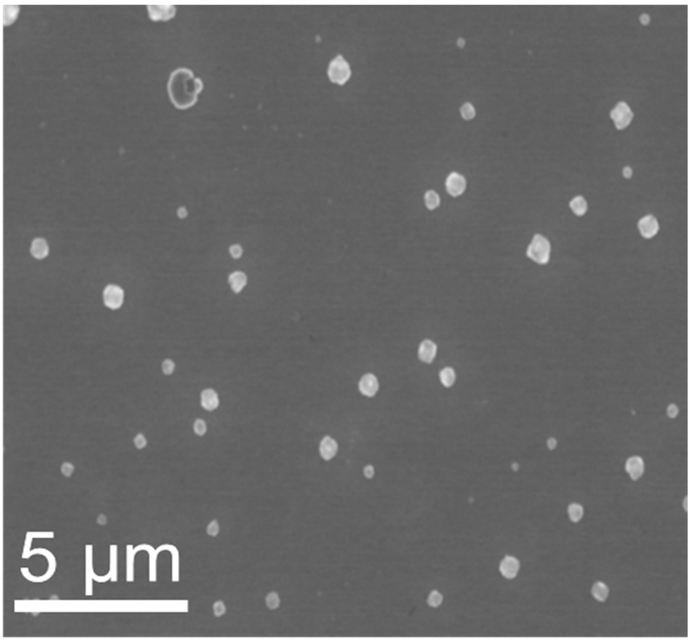

Figure S1. SEM image of TiO_2 nanoparticles at low magnification.

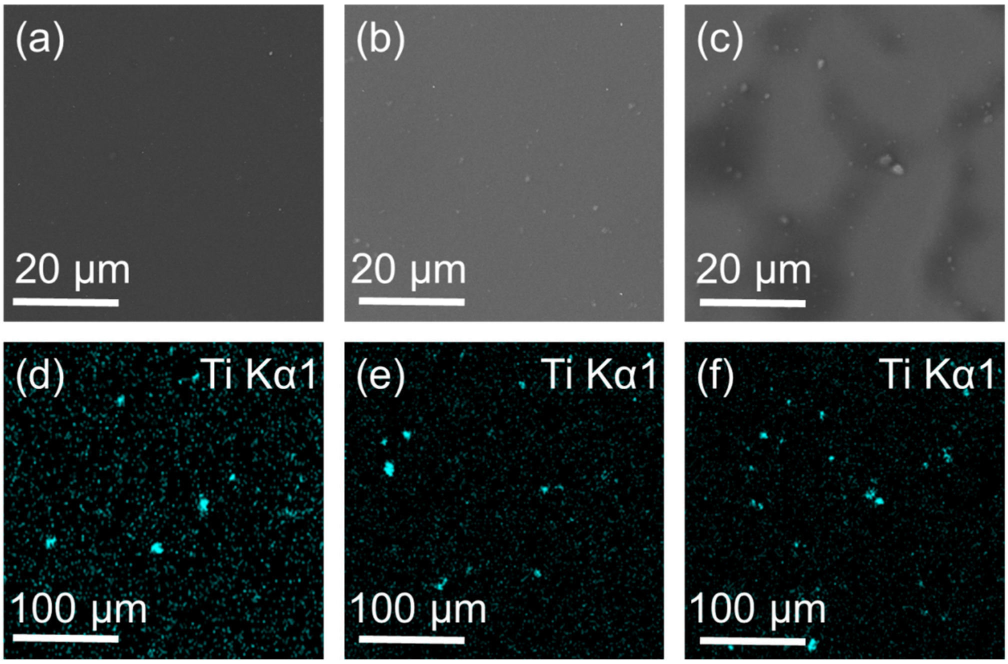

Figure S2. Characterization of TiO_2/PI nanocomposites. **(a)**, **(b)**, **(c)** SEM image and **(d)**, **(e)**, **(f)** corresponding respectively EDS mapping of TiO_2/PI nanocomposites with various loading contents of 1, 2, and 5 wt%.

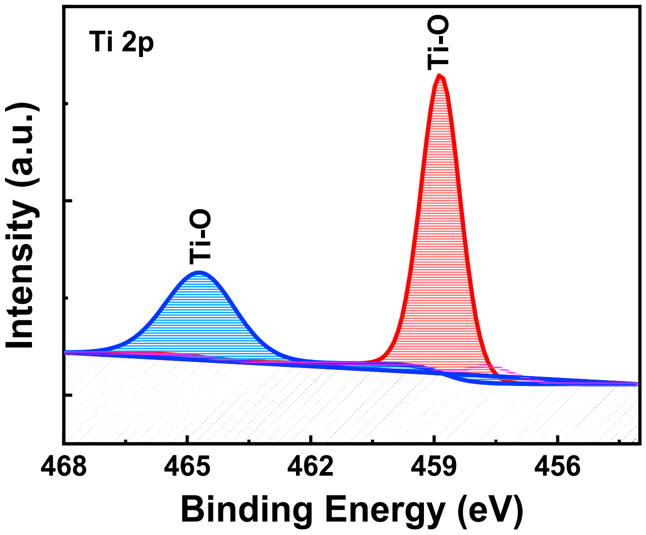

Figure S3. XPS spectra of Ti 2p components of TiO_2/PI nanocomposite.

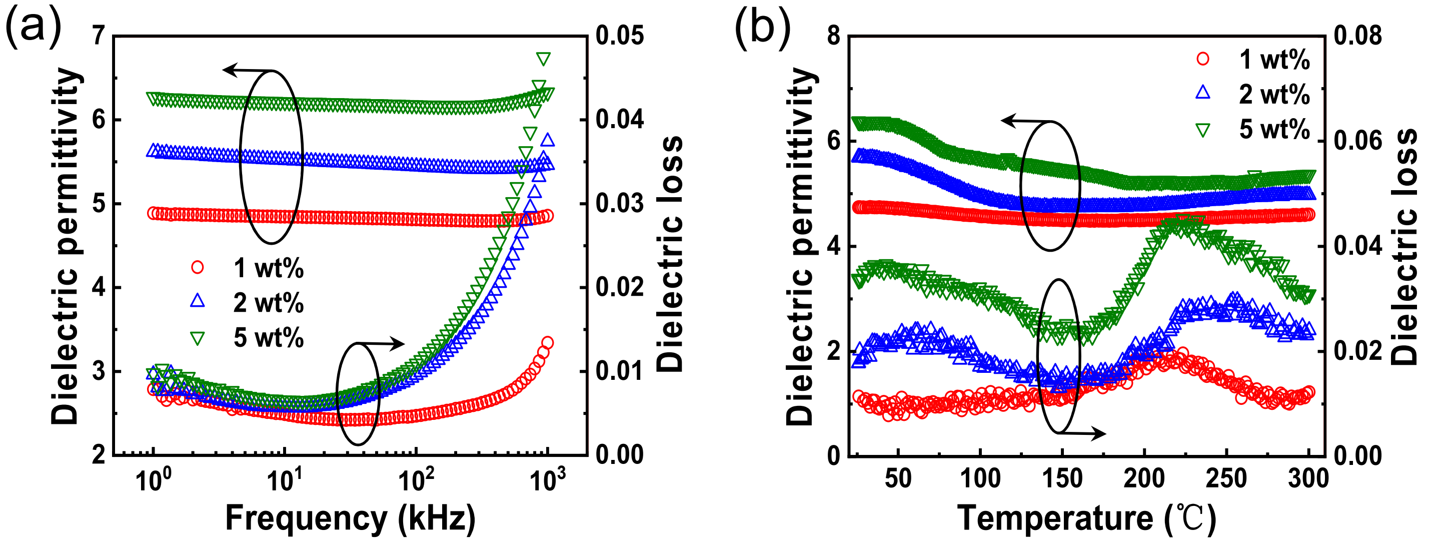

Figure S4. Dielectric properties of solid TiO_2/PI nanocomposites. Frequency-dependent **(a)** dielectric permittivity and dielectric loss of solid TiO_2/PI nanocomposites with various loading contents of 1, 2, and 5 wt% at RT. Temperature-dependent **(b)** dielectric permittivity and dielectric loss of solid TiO_2/PI nanocomposites with various loading contents of 1, 2, and 5 wt% at 1 kHz.

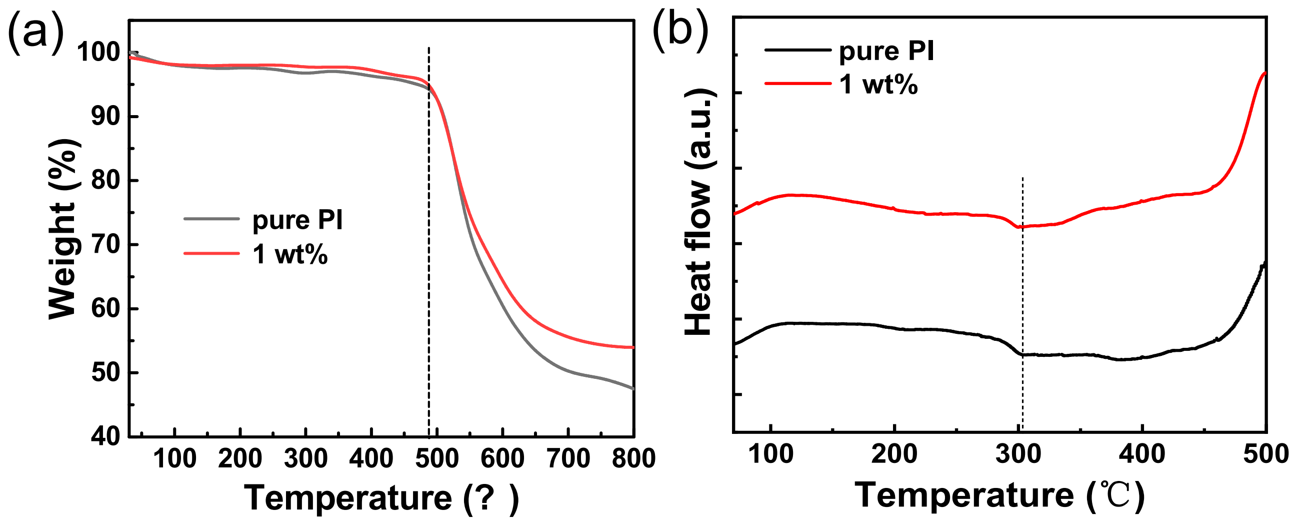

Figure S5. (a) TGA at the temperatures of 30 °C to 800 °C and **(b)** DSC curves at 100 °C to 500 °C of pure PI and TiO_2 nanoparticles/PI nanocomposite with 1 wt% content.

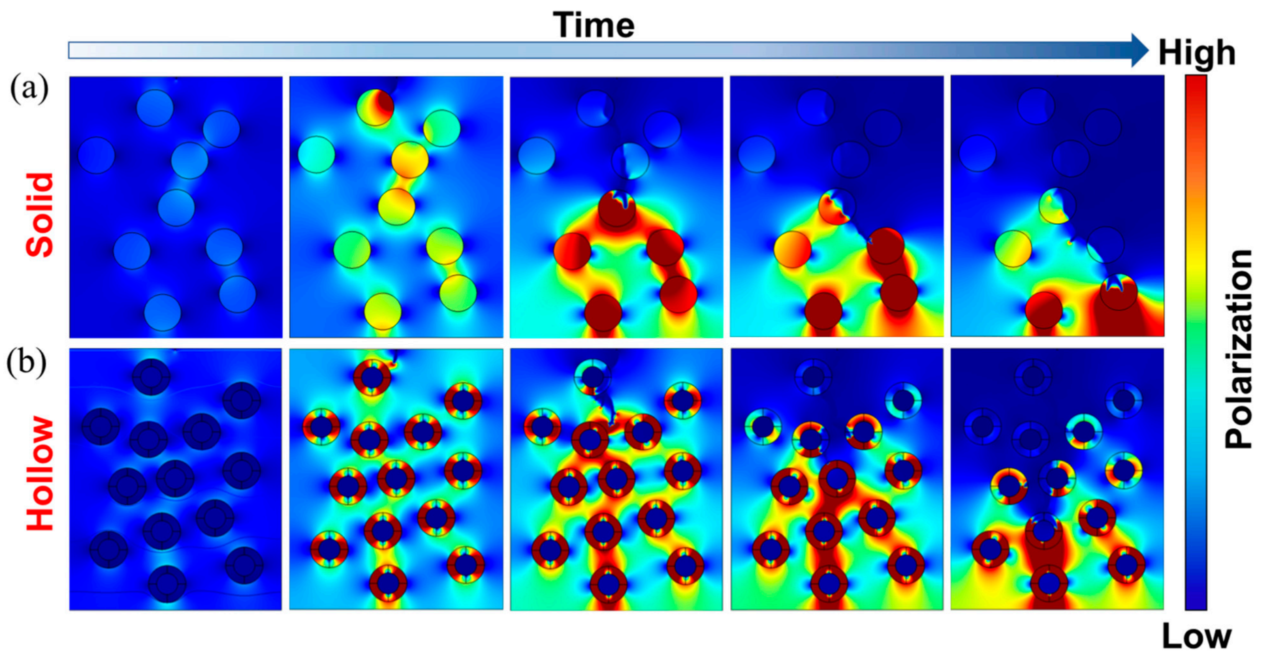

Figure S6. Finite element simulation of polarization evolution of TiO_2/PI nanocomposites with **(a)** solid and **(b)** hollow nanofillers.

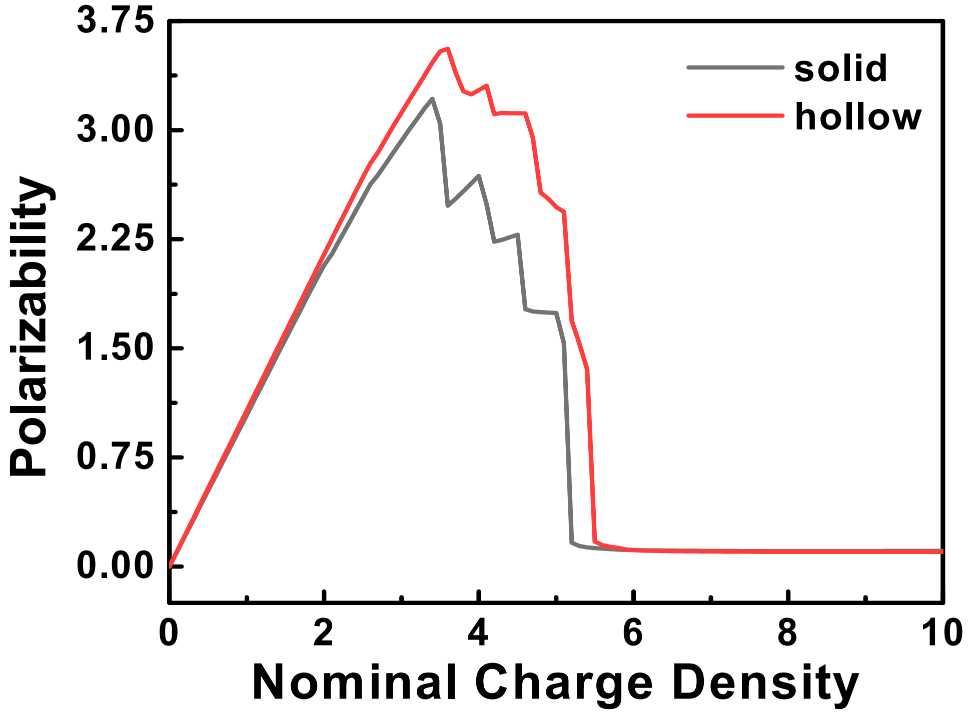

Figure S7. Relationship between nominal charge density and polarizability for PI-based nanocomposites with solid (grey curve) and hollow (red curve) nanoparticles.
